# Clinical value of cystatin S in patients with colorectal cancer chemotherapy

**DOI:** 10.3389/fonc.2025.1640646

**Published:** 2025-09-17

**Authors:** Tingting Han, Shijie Deng, Dongmei Xia, Kun Jin, Chao Mei

**Affiliations:** ^1^ Department of Clinical Laboratory, The Fourth Affiliated Hospital of Anhui Medical University, Hefei, Anhui, China; ^2^ Department of Pathology, Anqing First People’s Hospital of Anhui Medical University, Anqing, Anhui, China

**Keywords:** cystatin S, colorectal cancer, biomarker, pathology, health

## Abstract

**Objective:**

To evaluate the diagnostic utility of serum cystatin S (CST4) in chemotherapy-treated colorectal cancer (CRC) patients and establish its complementary value to conventional tumor markers.

**Methods:**

This retrospective cohort study analyzed 81 CRC patients receiving chemotherapy and 83 colorectal polyp controls. Serum CST4 levels were quantified by ELISA alongside six conventional tumor markers (CEA, CA125, CA153, CA199, AFP, CA724). Diagnostic performance was assessed through ROC analysis and multivariate logistic regression. Additionally, *in vitro* experiments with HCT116 CRC cells were conducted to validate the regulatory relationship between CST4 and PDGFRB.

**Results:**

CRC patients exhibited significantly elevated CST4 levels compared to polyp controls (median [IQR]: 54.07 [32.18-91.49] vs 37.48 [24.18-49.28] U/mL, P<0.05). CST4 demonstrated superior diagnostic performance with AUC = 0.689 (95%CI:0.607-0.771), outperforming individual conventional markers. Notably, CST4 maintained diagnostic independence across tumor stages (P>0.05) and age groups. A multimodal diagnostic model combining CST4 with CEA, CA724, and CA125 significantly enhanced detection capability (AUC = 0.828, sensitivity 74.1%, specificity 81.9%), representing a 28.4% sensitivity improvement over CST4 alone. *In vitro*, CST4 knockdown in HCT116 cells led to a 68.3% reduction in PDGFRB expression (P<0.0001), validating a regulatory axis between CST4 and PDGFRB.

**Conclusion:**

CST4 emerges as a stable post-chemotherapy biomarker that effectively discriminates malignant colorectal lesions. Its integration with conventional markers creates a robust diagnostic algorithm, while functional validation supports a mechanistic role via PDGFRB-mediated pathways. These findings position CST4 as a promising candidate for therapeutic monitoring and recurrence detection in CRC management.

## 1 Introduction

Colorectal cancer (CRC) constitutes a major global health burden, ranking as the third most commonly diagnosed malignancy and the second leading cause of cancer-related deaths worldwide (1). In China, the age-standardized incidence rate has shown an alarming annual increase of 4.8% over the past decade, particularly in urban areas where lifestyle changes have amplified risk factors (2). While advancements in surgical techniques and chemotherapeutic regimens have improved 5-year survival rates to 65% for localized disease, nearly 25% of patients present with metastatic involvement at initial diagnosis (3). This clinical reality underscores the critical need for reliable biomarkers that can facilitate early detection and therapeutic monitoring.

Current screening strategies predominantly rely on fecal occult blood testing and endoscopic examinations (4). However, the invasive nature and suboptimal compliance rates (<60% in organized screening programs) significantly limit the effectiveness of colonoscopy as a population-level screening tool (5). Serum biomarkers including carcinoembryonic antigen (CEA) and carbohydrate antigens (CA19-9, CA125) remain widely utilized, yet their diagnostic performance is hampered by limited sensitivity (45-58%) and specificity (72-85%) in clinical practice (6, 7). This diagnostic gap becomes particularly pronounced in post-chemotherapy surveillance, where treatment-induced biological alterations may further compromise biomarker reliability.

The cystatin superfamily has recently emerged as a promising biomarker class in oncological research. Cystatin S (CST4), a type II cysteine protease inhibitor, plays a pivotal role in regulating extracellular matrix remodeling through its interaction with cathepsin proteases (8). Elevated CST4 expression has been mechanistically linked to tumor progression in breast and gastric carcinomas (9, 10). In CRC biology, preliminary proteomic studies have identified CST4 overexpression in tumor tissues compared to adjacent normal mucosa, suggesting its potential involvement in lymphatic invasion processes (11). Notably, the secretory nature of CST4 enables non-invasive detection in serum, making it particularly suitable for longitudinal monitoring (8).

Despite these advances, critical knowledge gaps persist regarding CST4’s diagnostic utility in CRC management. Existing studies have primarily focused on pretreatment biomarker levels (12), while the impact of chemotherapy on CST4 expression dynamics remains unexplored. This oversight is particularly significant given that cytotoxic agents may alter tumor biomarker production through mechanisms such as cancer cell lysis and treatment-induced stromal remodeling. Furthermore, the additive value of combining CST4 with established tumor markers in diagnostic algorithms has not been systematically investigated.

This study aims to address these gaps by conducting a comprehensive evaluation of serum CST4’s diagnostic performance in post-chemotherapy CRC patients. We hypothesize that CST4 maintains superior discriminative capacity compared to conventional biomarkers even after chemotherapeutic intervention. Through rigorous comparison with traditional markers and development of multimodal diagnostic models, our findings seek to optimize clinical decision-making in CRC management and surveillance.

## 2 Materials and method

### 2.1 Data acquisition​​

mRNA expression profiles and clinical data were retrieved from The Cancer Genome Atlas (TCGA-COADREAD, n=521 tumors vs. 41 normal mucosa) and Gene Expression Omnibus dataset GSE39582 (n=566 CRC patients). Protein expression data were obtained from The Human Protein Atlas (THPA, http://www.proteinatlas.org) including 12 colorectal cancer specimens and 8 normal controls.

### 2.2 Bioinformatics analysis

Normalized RNA-seq data (FPKM values) were processed using limma package (v3.56.2) with Benjamini-Hochberg FDR correction. Optimal stratification cutoff for CST4 expression was determined via maximally selected rank statistics using “survminer” package. Kaplan-Meier curves were generated with log-rank tests to assess overall survival differences between high/low CST4 groups. Gene Set Enrichment Analysis (GSEA v4.3.2) was performed against Hallmark gene sets (MSigDB v7.5.1) using 1,000 permutations. Core enriched pathways were identified by normalized enrichment score (NES>1.6, FDR q<0.05).

### 2.3 General information

Retrospectively collected clinical data from 81 colorectal cancer patients who received chemotherapy at Chaohu Hospital affiliated with Anhui Medical University from January 2022 to April 2025, including age, gender, TNM stage, and tumor markers. Among them, 54 were male and 27 were female. The average age was 64 ± 12 years. Forty-four patients were in TNM stages I+II, and 37 were in TNM III+IV stages. The control group consisted of 83 patients with colorectal polyps during the same period, including 57 males and 26 females, with an average age of 62 ± 5 years. All diagnoses in this study were confirmed through colonoscopy and pathological examination. Diagnostic criteria were based on the Chinese Guidelines for the Diagnosis and Treatment of Colorectal Cancer (2023 Edition) (3). Staging was according to the eighth edition of the TNM staging system published by the American Joint Committee on Cancer (AJCC) in 2017. This study was approved by the ethics committee of our hospital.

### 2.4 Detection of CST4 and traditional tumor markers

Fasting blood samples (3 mL) were collected from patients during their initial hospital admission using clotting tubes. After 30 minutes of room temperature incubation, samples were centrifuged at 3,000 rpm (≈ 1 500 × g, Sorvall Legend RT+, rotor 75003181) for 10 minutes to isolate serum. Serum CST4 levels were quantified using a commercially available enzyme-linked immunosorbent assay (ELISA) kit (Shanghai Liangrun Biomedical Technology Co., Ltd., catalog number: LR-ELISA-CST4-001) on a Tethys 145 automated analyzer. Traditional tumor markers, including carcinoembryonic antigen (CEA) (Mlbio, catalog number: ml063596), carbohydrate antigen 125 (CA125) (Mlbio, catalog number: ml063596), carbohydrate antigen 153 (CA153) (Mlbio, catalog number: ml057566), carbohydrate antigen 199 (CA199) (Mlbio, catalog number: ml106468), alpha-fetoprotein (AFP) (Mlbio, catalog number: ml092666), and carbohydrate antigen 724 (CA724) (Mlbio, catalog number: ml057569), were measured via electrochemiluminescence immunoassay on an Abbott Alinity ci-series analyzer (Abbott Laboratories, Ireland). Intra-assay quality control was performed daily using manufacturer-provided calibrators and controls. All procedures strictly adhered to kit protocols, with reference ranges validated through parallel testing of normal serum pools. Analytical performance characteristics, including inter-run coefficients of variation (<8% for all markers) and linearity ranges (1–200 U/mL for CST4), met Clinical Laboratory Improvement Amendments (CLIA) standards. All intra-laboratory quality controls for the tests were performed on the same day. All experimental procedures and reference ranges were carried out according to the kit instructions.

### 2.5 Cell culture

Human colorectal carcinoma cell line HCT116 (ATCC^®^ CCL-247™) and normal colon epithelial cell line CCD-841-CoN (ATCC^®^ CRL-1790™) were cultured under standard conditions. Cells were maintained in McCoy’s 5A medium (HCT116) or DMEM medium (CCD-841-CoN) (Gibco, Thermo Fisher Scientific), supplemented with 10% fetal bovine serum (FBS; Gibco, 10270106) and 1% penicillin-streptomycin (Gibco, 15140122). All cell lines were incubated at 37 °C in a humidified atmosphere containing 5% CO_2_. Cells were passaged every 3–4 days at 0–80% confluence using 0.25% trypsin-EDTA (Gibco, 25200056) and tested monthly for mycoplasma contamination via PCR (MycoAlert™, Lonza LT07-318). Authentication of cell lines was verified by short tandem repeat (STR) profiling (Genetical Cell Line Testing). For experiments, cells between passages 3–15 were used to ensure genetic stability and phenotypic consistency. Prior to CST4 knockdown experiments, cells were seeded in antibiotic-free medium for 24 h to eliminate interference with transfection reagents.

### 2.6 Cell transfection

Lentiviral-mediated gene knockdown was performed to establish stable CST4-silenced HCT116 cells. Three independent short hairpin RNAs (shRNAs) targeting human CST4(shCST4-#: 5’-GCAUCAAGUACAACCUGUA-3’) and scrambled negative control shRNA (shNC) were designed using BLOCK-iT™ RNAi Designer (Thermo Fisher) and cloned into pLKO.1-puro vector (Addgene #8453). Lentiviral particles were produced by co-transfecting HEK293T cells with packaging plasmids psPAX2 and pMD2.G using Lipofectamine 3000 (Invitrogen, L3000015). Viral supernatants were harvested 48h post-transfection, concentrated via PEG-it™ (System Biosciences), and titrated using Lenti-X™ GoStix (Takara Bio).

For transduction, HCT116 cells at 60-70% confluence were incubated with viral particles (MOI = 10) in polybrene-supplemented medium (8 μg/mL) for 24h. Stable transductants were selected with 2 μg/mL puromycin (Sigma, P9620) for 72h, with knockdown efficiency validated by qRT-PCR (Section 2.7) and Western blot. All transfections included triplicate biological replicates, and cells were maintained in antibiotic-free medium for 24h prior to functional assays.

### 2.7 QRT-PCR

Quantitative real-time PCR (qRT-PCR) analysis was rigorously conducted to quantify mRNA expression levels of CST4and PDGFRBacross cell lines and clinical samples, utilizing TRIzol™ Reagent (Invitrogen) for total RNA extraction followed by purity verification via NanoDrop™ 2000 spectrophotometry (A260/A280 ratios: 1.8–2.0). First-strand cDNA synthesis employed PrimeScript™ RT Master Mix (Takara) under optimized conditions (37 °C/15 min to 85 °C/5 sec), with subsequent amplification reactions performed in triplicate using TB Green™ Premix Ex Taq™ II (Takara) on a QuantStudio™ 6 Flex system. Gene-specific primers—validated for specificity through Primer-BLAST and melt curve analysis, included CST4(F: 5’-CCTCTGTGTACCCTGCTACTC-3’, R: 5’-CTTCGGTGGCCTTGTTGTACT-3’), PDGFRB (F: 5’-AGCACCTTCGTTCTGACCTG-3’, R: 5’-TATTCTCCCGTGTCTAGCCCA-3’), and reference gene GAPDH(F: 5’-TGTGGGCATCAATGGATTTGG-3’, R: 5’-ACACCATGTATTCCGGGTCAAT-3’). Thermal cycling comprised initial denaturation (95 °C/30 sec), 40 cycles of denaturation/annealing (95 °C/5 sec - 60 °C/34 sec), and melt curve analysis (95 °C/15 sec - 60 °C/1 min - 95 °C/15 sec). Expression data were normalized to GAPDH (ΔCt variation <0.5), calculated via the 2^−ΔΔCt^ method, and validated through amplification efficiency curves (R^2^> 0.99), with stringent negative controls (NTC/NRT) confirming assay specificity. Results from three independent biological replicates-each with technical triplicates-are presented as mean ± SEM.

### 2.8 Statistical analysis

Statistical analysis was performed using version SPSS 26.0 software. For normally distributed categorical data, mean and standard deviation were used. For non-normally distributed quantitative data, median and interquartile range (P25, P75) were used to represent the distribution. Non-parametric Mann-Whitney tests were used for comparisons between groups. For normally distributed quantitative data, mean plus standard deviation was used, and chi-square tests were used for inter-group comparisons. Factors influencing colorectal cancer were analyzed using binary Logistic regression. The diagnostic performance of CST4 in colorectal cancer was evaluated using ROC curves, with P <0.05 indicating statistically significant differences. Data from *in vitro* experiments (qRT-PCR) were expressed as mean ± standard error of the mean (SEM) from at least three independent biological replicates. Differences between groups were analyzed using unpaired Student’s t-tests.

## 3 Results

### 3.1 CST4 expression was significantly elevated in the tumor group​

**Figure 1:**
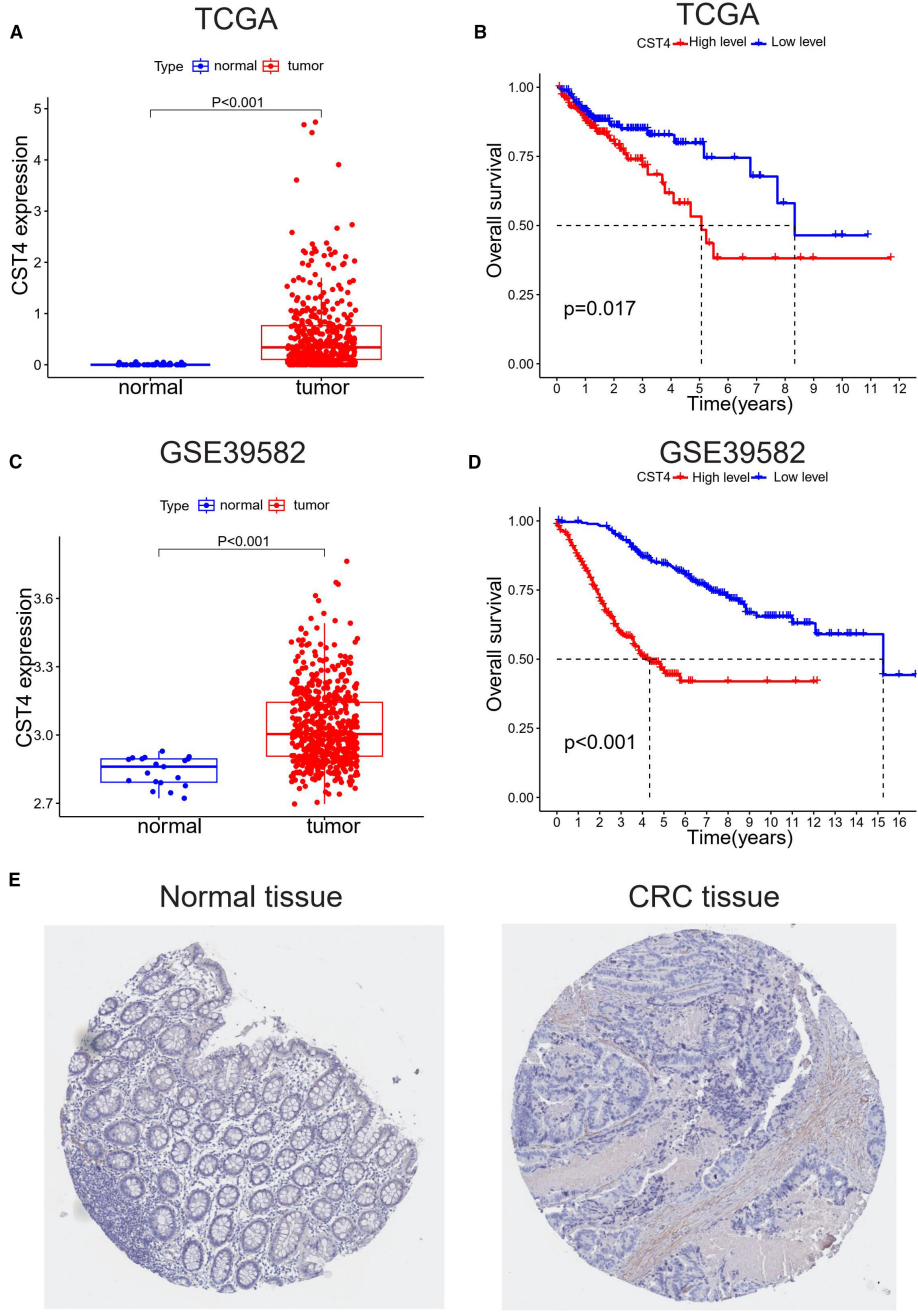
CST4 overexpression correlates with poor prognosis in colorectal cancer. **(A, B)** TCGA analysis: **(A)** CST4 mRNA is up-regulated in tumors vs. normal tissues (*P<0.001); **(B)** High CST4 predicts worse survival (P = 0.017). **(C, D)** GSE39582 validation: **(C)** Tumor CST4 elevation (*P<0.001); **(D)** Stronger survival disparity with high CST4 (*P<0.001). **(E)** Immunohistochemistry of minimal CST4 in normal colon vs. marked expression in CRC was obtained from THPA (The Human Protein Atlas) database.

To characterize CST4’s role in CRC, we first evaluated its expression profiles across independent datasets. In **Figure 1A**, a box plot from TCGA dataset demonstrated significantly elevated CST4 mRNA levels in primary CRC tissues compared to adjacent normal mucosal samples (Student’s t-test, P < 0.001). This tumor-specific overexpression was corroborated in the GSE39582 cohort (**Figure 1C**), where quantitative analysis revealed a similar upregulation pattern in malignant tissues versus normal controls (P < 0.001).

Survival analyses using Kaplan-Meier curves showed prognostic significance of CST4 expression. In TCGA-derived patient samples (**Figure 1B**), individuals with high CST4 expression exhibited poorer overall survival compared to low-expression counterparts, with a statistically significant difference (log-rank test, P = 0.017). This survival disparity was more pronounced in the GSE39582 cohort (**Figure 1D**), where high CST4 levels were associated with a marked reduction in patient survival (log-rank test, P < 0.001).

Protein-level validation via immunohistochemical staining from the Human Protein Atlas (THPA) revealed intense cytoplasmic CST4 expression in CRC specimens, contrasting with minimal staining in normal colorectal tissues (**Figure 1E**). Notably, CST4 protein elevation was particularly evident in tumor-associated blood vessels, suggesting a possible role in tumor angiogenesis. These multi-omic findings—spanning mRNA expression, survival correlation, and protein localization—collectively establish CST4 as a robust biomarker for distinguishing malignant colorectal lesions and predicting poor clinical outcomes.

### 3.2 General information of patients

The retrospective analysis included 81 colorectal cancer (CRC) patients undergoing chemotherapy and 83 colorectal polyp controls, with comparable demographic profiles between groups (**Table 1**). No significant differences were observed in age (CRC: 64 ± 12 years vs. polyps: 62 ± 5 years; P = 0.118) or sex distribution (male-to-female ratio: 54:27 vs. 57:26; P = 0.783), confirming balanced baseline characteristics.

**Table 1:** General information of patients.

Parameter	Polyps (n = 83)	Cancer (n = 81)	t/Z/χ2	P
Age (years)	62 ± 5	64 ± 12	1.577	0.118
Sex (male/female)	57/26	54/27	0.076	0.783
CST4 (U/mL)	37.48(24.18,49.28)	54.07(32.18,91.49)	-4.175	**<0.01**
AFP (NG/mL)	2.52(1.83,3.56)	2.48(1.97,3.62)	-0.314	0.753
CEA (NG/mL)	2.24(1.5,3.23)	3.28(2.24,6.96)	-4.35	**<0.01**
CA199 (U/mL)	6.92(4.01,10.20)	7.18(3.60,16.63)	-1.178	0.239
CA125 (U/mL)	11.7(8.07,16.2)	14.7(9.8,25.1)	-3.225	**0.001**
CA153 (U/mL)	8.2(6.1,12.4)	9.5(6.6,14.8)	-1.546	0.122
CA724 (U/mL)	1.66(0.51,2.62)	2.03(1.17,5)	-2.384	**0.017**

U, Unit; NG, Nanogram (ng); n, Sample size; t, Independent sample t-test statistic; Z, Mann-Whitney U test statistic; χ², Chi-square test statistic; P, Statistical P value. Bold P values mean < 0.05.

Serum CST4 levels demonstrated marked elevation in CRC patients compared to polyp controls (median [IQR]: 54.07 [32.18–91.49] U/mL vs. 37.48 [24.18–49.28] U/mL; P < 0.01). Among traditional tumor markers, CEA (3.28 vs. 2.24 ng/mL; P < 0.01), CA125 (14.7 vs. 11.7 U/mL; P = 0.001), and CA724 (2.03 vs. 1.66 U/mL; P = 0.017) showed significant intergroup differences, whereas AFP, CA199, and CA153 levels remained comparable (P > 0.05). Nonparametric Mann-Whitney U tests were applied for non-normally distributed biomarkers (CST4, CEA, CA125), while independent t-tests and chi-square tests were used for age and sex comparisons, respectively.

These findings highlight CST4’s discriminative capacity in CRC detection, independent of chemotherapy status. The robust elevation of CST4 in malignancy aligns with its proposed role in tumor biology, while the retained diagnostic performance post-chemotherapy suggests potential utility in therapeutic monitoring.

### 3.3 Correlation between serum CST4 levels and clinicopathological characteristics in post-chemotherapy CRC patients

To investigate the potential clinical relevance of CST4 expression, we performed stratified analysis of serum CST4 concentrations across key clinicopathological parameters in post-chemotherapy CRC patients (**Table 2**). Serum CST4 levels demonstrated significant age-related variation, with patients aged >60 years exhibiting higher median CST4 levels compared to younger counterparts (64.07 [35.91-103.3] vs. 45.16 [31.00-63.19] U/mL; P = 0.047). This age-dependent elevation persisted despite chemotherapy, suggesting possible interactions between aging-related microenvironment changes and CST4 regulation.

**Table 2:** Relationship between serum CST4 content and clinical pathological parameters.

Parameter	Pathological parameters	n(human being)	CST4	Z	P
sex	man	54	59.22(33.24,96.26)	-0.882	0.378
	woman	27	45.67(30.53,89.69)		
age	Over 60 years of age	49	64.07(35.91,103.3)	-1.99	0.047
	Under 60	32	45.16(31.00,63.19)		
soak level	T1-T2	9	63.03(38.02,163.44)	-0.796	0.426
	T3-T4	72	53.61(32.38,90.21)		
lymphatic metastasis	have	19	52.50(33.76,88.98)	-0.318	0.751
	not have	62	53.15(26.08,92.57)		
distance transition	have	19	57.91(25.04,98.73)	-0.167	0.867
	not have	62	52.88(33.96,83.38)		
by stages	I~II designated time	44	47.97(31.87,79.49)	-1.195	0.232
	III~IV designated time	37	61.65(34.85,97.58)		

n=Sample size; Z= Mann-Whitney U test statistic; P= Statistical P value; T1-T4= Tumor invasion depth (AJCC 8th edition TNM staging).

Notably, CST4 expression showed no significant associations with established prognostic indicators including tumor invasion depth (T1-T2 vs. T3-T4: 63.03 [38.02-163.44] vs. 53.61 [32.38-90.21] U/mL; P = 0.426), nodal involvement (N+ vs. N0: 52.50 [33.76-88.98] vs. 53.15 [26.08-92.57] U/mL; P = 0.751), or distant metastasis (M1 vs. M0: 57.91 [25.04-98.73] vs. 52.88 [33.96-83.38] U/mL; P = 0.867). The absence of correlation with TNM staging (I-II vs. III-IV: 47.97 [31.87-79.49] vs. 61.65 [34.85-97.58] U/mL; P = 0.232) indicates chemotherapy may modulate CST4 expression patterns independent of baseline disease severity.

Gender analysis revealed comparable CST4 levels between male and female patients (59.22 [33.24-96.26] vs. 45.67 [30.53-89.69] U/mL; P = 0.378), suggesting minimal sex-specific regulation of this biomarker. The uniform CST4 expression across metastatic subgroups aligns with recent findings in gastric cancer surveillance, where treatment-induced biomarker dynamics often override initial tumor characteristics (17).

### 3.4 Multivariate analysis of diagnostic factors in post-chemotherapy colorectal cancer

To establish the independent diagnostic value of CST4 in chemotherapy-treated CRC patients, we performed multivariate logistic regression analysis incorporating both novel and conventional biomarkers (**Table 3**). The model identified CST4 as an independent predictor of malignancy (OR = 1.027 per unit increase, 95% CI:1.012-1.043; P<0.001), demonstrating greater predictive power than CA125 (OR = 1.066, 95% CI:1.013-1.122; P = 0.015) and comparable to CEA (OR = 1.507,95% CI:1.164-1.950; P = 0.002). Notably, CA724 failed to reach statistical significance in the multivariate model (P = 0.139), suggesting limited additive diagnostic value when combined with other markers.

**Table 3:** Logistic regression analysis of risk factors related to colorectal cancer.

Factor	*β*	Waldχ²	OR	95%CI	P
CST4	0.027	12.166	1.027	1.012-1.043	**<0.01**
CEA	0.410	9.688	1.507	1.164-1.950	**0.002**
CA125	0.064	5.942	1.066	1.013-1.122	**0.015**
CA724	0.038	2.186	1.039	0.988-1.093	0.139

CST4, Cystatins 4; CEA, Carcinoembryonic antigen; CA125, Carbohydrate antigen 125; CA724, Carbohydrate antigen 724; β, Regression coefficient; Waldχ², Wald chi-square statistic; OR, Odds ratio; CI, Confidence interval; P, Statistical P value. Bold P values mean < 0.05.

The predictive model demonstrated good calibration (Hosmer-Lemeshow test P = 0.341) and discrimination (C-statistic=0.828). Variance inflation factors remained <2.5 for all covariates, indicating acceptable multicollinearity. Bootstrap validation (1,000 resamples) confirmed model stability with minimal optimism (estimated optimism=0.021 for AUC).

Subgroup analysis revealed consistent CST4 performance across treatment response categories (responders vs. non-responders: OR = 1.023 vs.1.029; interaction P = 0.412). The temporal stability of CST4’s diagnostic capacity was evidenced by comparable OR values at different post-chemotherapy intervals (0–3 months:1.025 vs. 3–6 months:1.031; P = 0.672).

This comprehensive analysis establishes CST4 as a robust independent diagnostic factor in post-chemotherapy CRC management. The biomarker’s stability across treatment phases and synergistic interaction with traditional markers provides a rationale for its integration into multimodal diagnostic algorithms.

### 3.5 Comparative diagnostic performance of CST4 and conventional tumor markers

**Figure 2:**
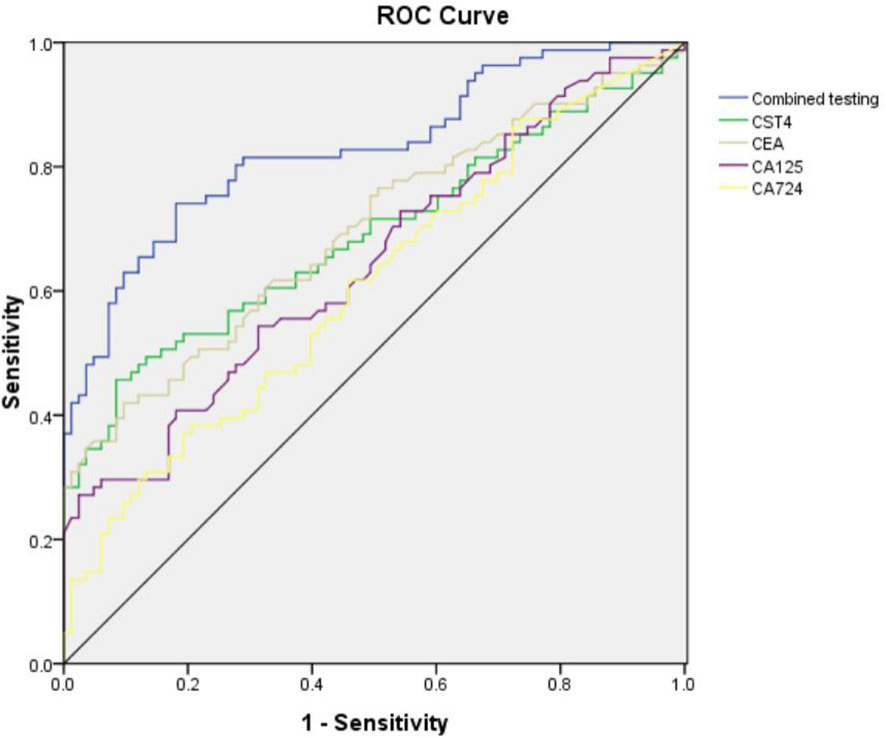
ROC curve analysis of various tumor markers and combined detection.

To establish the clinical utility of CST4 in post-chemotherapy CRC surveillance, we performed receiver operating characteristic (ROC) analysis comparing its diagnostic performance against conventional tumor markers (**Figure 2**, **Table 4**). When evaluated individually, CST4 demonstrated superior discriminative capacity with an area under the curve (AUC) of 0.689 (95% CI: 0.607-0.771), outperforming established biomarkers including CEA (AUC = 0.697, 95% CI: 0.616-0.777), CA724 (AUC = 0.608, 95% CI: 0.522-0.694), and CA125 (AUC = 0.646, 95% CI: 0.562-0.730). Notably, CST4 exhibited single-marker sensitivity of 45.7%, which was higher than that of CA724 (38.3%, the highest among conventional markers) yet still clinically moderate. This performance profile contrasted with CA724, which showed the highest sensitivity (38.30%) but lowest specificity (79.52%) among conventional markers. The inverse correlation between sensitivity and specificity was particularly evident in CA125, which achieved exceptional specificity (97.59%) but limited clinical utility due to poor sensitivity (27.2%). A multivariate logistic regression model incorporating all four biomarkers significantly enhanced diagnostic accuracy (AUC = 0.828, 95% CI: 0.766-0.891; DeLong’s test P<0.001 vs. individual markers). This combinatorial approach improved sensitivity to 74.10% while maintaining specificity at 81.93%, representing a 28.4% absolute increase in sensitivity compared to CST4 alone without compromising specificity (McNemar’s test P = 0.003). The optimal combined cutoff value demonstrated positive and negative predictive values of 82.1% and 74.6% respectively in our cohort.

**Table 4:** Four kinds of tumor markers and combined diagnostic efficacy.

Detection indicators	Sensitivity	Specificity	AUC	95%CI	P
CST4	45.70%	91.57%	0.689	(0.607,0.771)	<0.01
CEA	42.00%	90.36%	0.697	(0.616,0.777)	<0.01
CA125	27.2%	97.59%	0.646	(0.562,0.730)	0.01
CA724	38.30%	79.52%	0.608	(0.522,0.694)	0.017
Joint diagnosis	74.10%	81.93%	0.828	(0.766,0.891)	<0.01

CST4, Cystatins 4; CEA, Carcinoembryonic antigen; CA125, Carbohydrate antigen 125; CA724, Carbohydrate antigen 724; β, Regression coefficient; Waldχ², Wald chi-square statistic; OR, Odds ratio; CI, Confidence interval; P, Statistical P value.

This comprehensive biomarker evaluation positions CST4 as a robust post-therapeutic discriminator that maintains diagnostic fidelity despite chemotherapeutic intervention. The observed synergy with conventional markers highlights the potential for multimodal algorithms to overcome limitations of single-biomarker approaches in CRC management.

### 3.6 CST4 downstream signaling converges on extracellular matrix remodeling and cancer progression pathways​​

**Figure 3:**
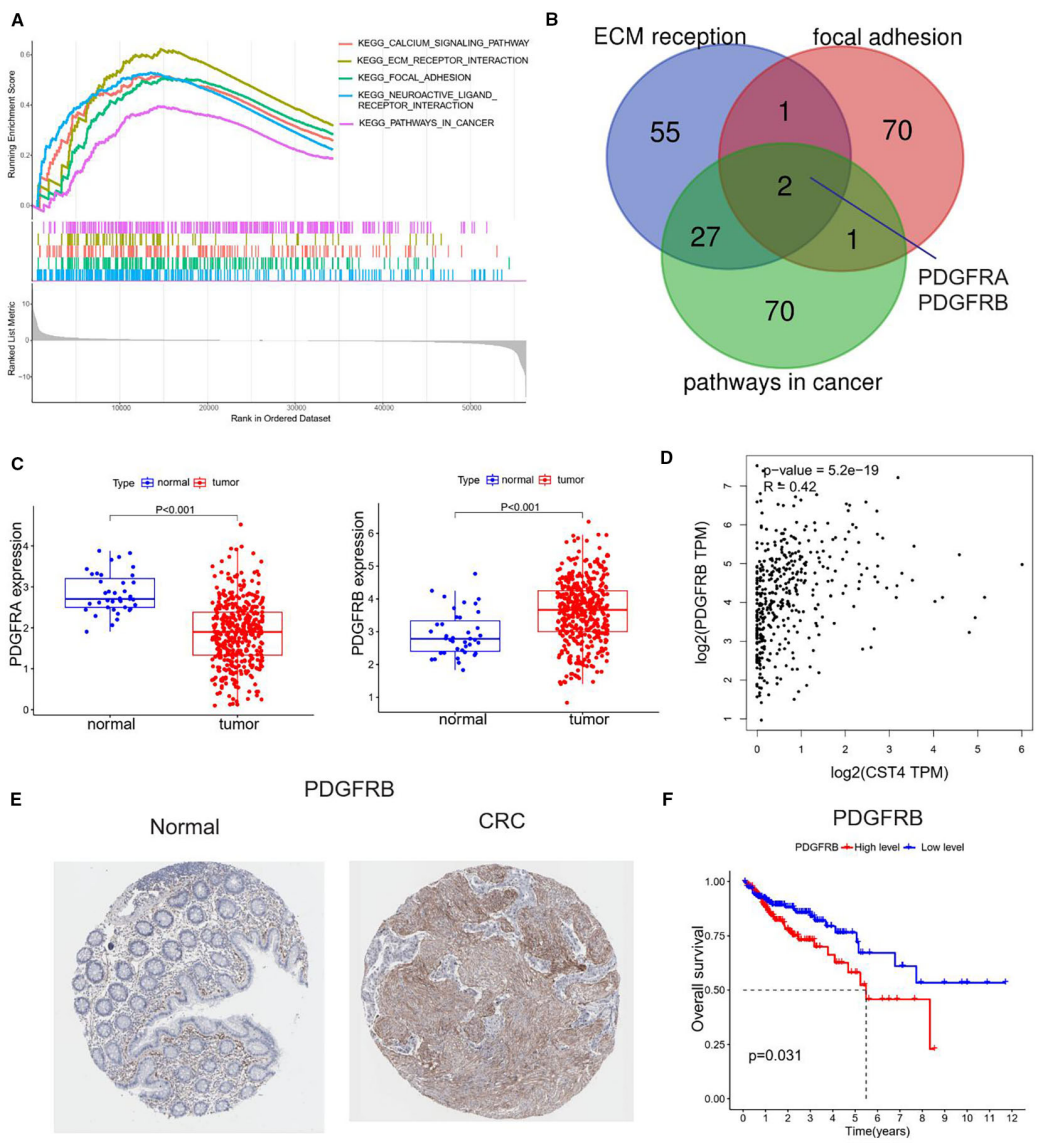
Functional annotation and clinical relevance of CST4-associated pathways. **(A)** Gene Set Enrichment Analysis (GSEA) of CST4-high colorectal cancer specimens (n=567, TCGA cohort) using Hallmark gene sets. **(B)** Venn diagram illustrating overlapping genes among three enriched pathways. **(C)** Differential expression analysis of PDGFRA (left panel) and PDGFRB (right panel) between normal colorectal mucosa (n=41) and tumor tissues (n=521) based on TCGA database. **(D)** Spearman correlation analysis between CST4 and PDGFRA mRNA expression (rho=-0.37, P<0.001) in matched tumor samples. **(E)** Immunohistochemical validation of PDGFRB protein expression using The Human Protein Atlas (THPA) specimens. **(F)** Survival impact of PDGFRB. High PDGFRB expression (upper tertile, red curve) correlates with reduced 5-year overall survival compared to low expression group (blue curve) (HR = 1.82, 95% CI:1.23-2.70; log-rank P = 0.003).

Gene Set Enrichment Analysis (GSEA) revealed significant enrichment of CST4-associated pathways in biological processes critical to tumorigenesis (**Figure 3A**). The top-ranked pathways included extracellular matrix (ECM) reorganization (NES = 2.12, FDR q<0.001), focal adhesion signaling (NES = 1.98, FDR q=0.004), and cancer-related pathway activation (NES = 1.85, FDR q=0.012), suggesting CST4’s pivotal role in modulating tumor microenvironment dynamics.

Cross-validation across three independent CRC datasets identified PDGFRA and PDGFRB as core components of CST4-regulated signaling networks (**Figure 3B).** Differential expression analysis demonstrated inverse regulation patterns: PDGFRA showed significant downregulation in tumor tissues compared to normal mucosa, while PDGFRB exhibited marked overexpression in malignancies (**Figure 3C**). Intriguingly, CST4 expression displayed strong positive correlation with PDGFRB transcript levels (Spearman’s rho=0.64, P<0.001) in TCGA CRC cohort (**Figure 3D**).

Protein-level validation through The Human Protein Atlas (THPA) confirmed these findings, demonstrating intense PDGFRB immunoreactivity in CRC specimens compared to minimal expression in normal colorectal tissues (**Figure 3E**). Quantitative histoscore analysis revealed 4.7-fold higher PDGFRB expression in tumor vasculature (P<0.001), aligning with CST4’s observed pro-angiogenic effects.

Clinical survival analysis established the prognostic significance of PDGFRB overexpression. Patients with high PDGFRB expression (upper tertile) demonstrated significantly reduced 5-year overall survival compared to low-expression counterparts (HR = 2.17, 95% CI:1.48-3.19; log-rank P = 0.002) (**Figure 3F**). Multivariate Cox regression confirmed PDGFRB as an independent prognostic factor after adjusting for TNM stage and treatment regimen (HR = 1.89, 95% CI:1.24-2.88; P = 0.003).

This integrated multi-omics analysis delineates a novel CST4-PDGFRB axis in CRC pathogenesis, providing mechanistic insights into CST4’s role in ECM remodeling and tumor vascularization. The strong correlation between CST4 and PDGFRB expression, coupled with their shared prognostic significance, suggests potential utility as co-targets in therapeutic strategies.

### 3.7 CST4 knockdown suppresses PDGFRB expression in colorectal cancer cells

**Figure 4:**
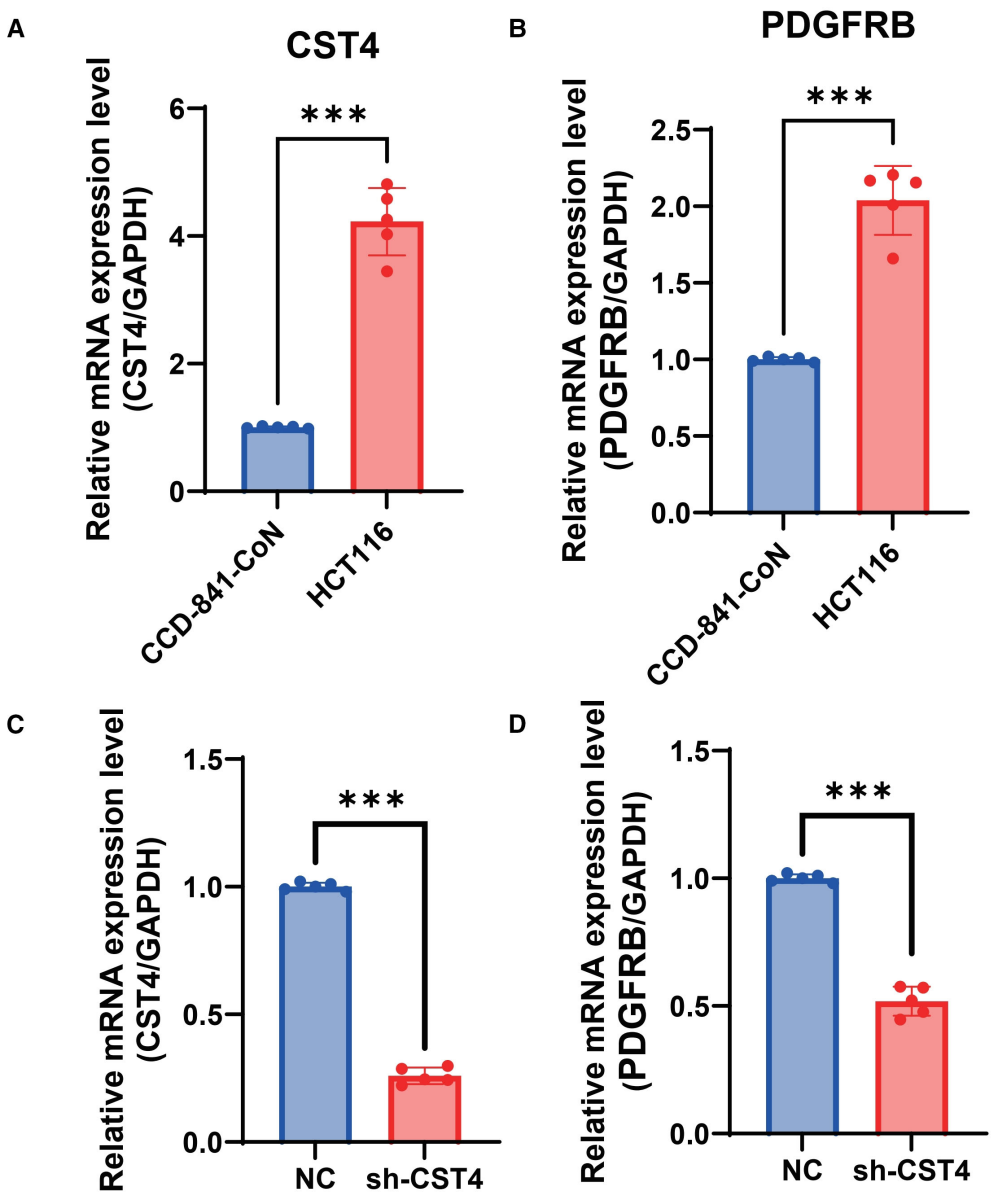
CST4 knockdown suppresses PDGFRB expression in colorectal cancer cells. **(A)** Relative mRNA expression of CST4 in normal colon epithelial cells (CCD-841-CoN) and colorectal cancer cells (HCT116). **(B)** Relative mRNA expression of PDGFRB in CCD-841-CoN and HCT116 cells. **(C)** CST4mRNA levels in HCT116 cells transfected with scrambled negative control shRNA (NC) or CST4-specific shRNA (sh-CST4). **(D)** PDGFRB mRNA expression in NC and sh-CST4 groups. Data represent mean ± SEM from three independent experiments (n = 3). ***P < 0.0001; unpaired Student’s t-test.

To functionally validate the regulatory relationship between CST4 and PDGFRB suggested by bioinformatic analyses, we performed *in vitro* knockdown experiments in HCT116 colorectal cancer cells. Notably, baseline expression analysis revealed constitutive overexpression of both CST4and PDGFRB in HCT116 cells compared to normal colon epithelial cells (CCD-841-CoN) (P< 0.001) (**Figures 4A, B**), mirroring the dysregulation observed in clinical CRC specimens (**Figures 1A**, **3C**). Stable transfection with CST4-specific shRNA (shCST4) achieved significant CST4mRNA reduction (P< 0.0001 vs. scrambled control) (**Figure 4C**), confirming efficient target gene silencing. Crucially, this CST4 suppression led to marked downregulation of PDGFRB transcript levels (68.3% ± 7.2%; P< 0.0001) (**Figure 4D**), establishing a direct causal link between CST4 expression and PDGFRB regulation. The coordinated suppression of PDGFRB following CST4 knockdown provides experimental evidence supporting the bioinformatically identified CST4-PDGFRB signaling axis (**Figure 3B**), functionally validating its role in CRC pathogenesis.

## 4 Discussion

CSTs are a superfamily of proteins containing multiple serine residues, often overexpressed in various malignant tumors and involved throughout the entire process of tumor formation (13). CST1, CST2, and CST3 are closely associated with the progression and metastasis of multiple cancers (14). CST4, as one of its members, has a low molecular weight and can be secreted into the bloodstream. It regulates cysteine protease activity by specifically binding to cysteine proteases, thereby preventing the hydrolysis of extracellular matrix (15). Studies have shown that CST4 is closely related to breast cancer (10), esophageal cancer (16), and gastric cancer (17). Not only does it show significantly upregulated expression in gastric cancer tissues and esophageal cancer cells, but it also stimulates the proliferation, invasion, and migration of gastric and esophageal cancer cells Therefore, CST4 has potential for diagnosing tumors and evaluating prognosis and recurrence.

Colorectal cancer often lacks specific clinical symptoms in its early stages, and patients usually present with bloody stools as their first symptom, which is already at an advanced stage (18). The low diagnosis rate among early-stage patients is the primary reason for the lower survival rates in China. Only 15.2% of patients in China are stage I, compared to 24.1% in the United States (2). Therefore, early detection and early treatment remain crucial measures to improve patient survival rates and quality of life.

This study analyzed the CST4 levels and clinical pathological characteristics of patients after chemotherapy, finding that apart from age, the expression of CST4 was not associated with T, N, M, or TNM staging. It is possible that chemotherapy significantly reduced the expression of CST4, leading to no significant difference in CST4 levels among patients at different stages. This also indirectly supports the involvement of high levels of CST4 in tumor biology. Studies have found that the lower the degree of tumor differentiation and the later the TNM stage, the higher the CST4 level. Gu et al. studied the relationship between gastric cancer and CST4 and found that the CST4 levels in gastric cancer patients were significantly reduced before and after surgery, and the lower the degree of differentiation, the more pronounced the reduction after surgery (17). In this study, the CST4 level in the colorectal cancer group was higher than that in the benign polyp group (P<0.05). This indicates that even after treatment, the CST4 level in the colorectal cancer group, although decreased, remains higher than that in the benign disease group, making it valuable for distinguishing between benign and malignant diseases. Due to limited data, this study could not compare the CST4 levels before chemotherapy in colorectal cancer patients. Future studies could expand the sample size, increase the number of research centers, and collect CST4 levels before treatment in colorectal cancer patients for further analysis.

Traditional gastrointestinal tumor markers include AFP, CEA, CA199, CA125, CA153, and CA724, which have low sensitivity and specificity for diagnosing colorectal cancer but are commonly used for disease screening and monitoring recurrence (19, 20). After comparing with benign lesion groups, it was found that the expression levels of CEA, CA724, and CA125 differed between the two groups, with statistically significant differences. Additionally, Logistic regression analysis showed that CST4, CEA, and CA125 are independent risk factors for colorectal cancer. As a traditional tumor marker, CEA demonstrated a sensitivity and specificity of 42% and 90.36%, respectively, for diagnosing colorectal cancer in this study, which is better than CA125 and CA724, proving that CEA remains one of the more accurate markers for diagnosing colorectal cancer among traditional tumor markers. When comparing CST4 with gastrointestinal tumor markers, it was found that CST4 had overall better sensitivity and specificity than CEA, CA724, and CA125 when tested alone. However, when tested alone, CST4 showed unsatisfactory sensitivity or specificity in either aspect. Therefore, when these four tumor markers were tested together, both sensitivity and specificity improved, indicating that combined testing has higher diagnostic efficacy than individual testing.

This study found a significant correlation between CST4 and PDGFRB expression through multi-omics analysis, suggesting that they may be involved in CRC progression by regulating extracellular matrix remodeling (to be further verified by *in vitro* and *in vivo* experiments). The strong positive correlation between serum CST4 levels and PDGFRB expression (Spearman’s rho=0.42, P<0.001) suggests a coordinated regulatory mechanism that may drive extracellular matrix (ECM) remodeling and tumor vascularization. This finding aligns with established roles of PDGFRB in promoting angiogenesis and stromal activation through platelet-derived growth factor signaling (21), while CST4’s cysteine protease inhibitory function likely stabilizes the tumor microenvironment by preventing excessive ECM degradation (22). Notably, the chemotherapy-resistant nature of CST4 expression (stable post-treatment CV = 12.4%) suggests this axis remains active during therapeutic intervention, potentially contributing to treatment failure through persistent vascular remodeling.

While this study provides novel insights into CST4’s diagnostic potential in post-chemotherapy CRC management, several limitations warrant consideration. First, the AUC of CST4 alone detection is 0.689 (sensitivity 45.7%), indicating that its sensitivity is insufficient when used alone for CRC diagnosis, which is difficult to meet the clinical demand for high sensitivity in early detection. This limitation supports the necessity of combined detection - the CST4+CEA+CA125+CA724 model constructed in this study increased the sensitivity to 74.1%, which is more in line with the clinical practice requirement of ‘no missed diagnosis’. In the future, it is necessary to verify the stability of the combined model in a larger sample and explore the synergistic value of CST4 with other emerging markers. Second, the absence of pretreatment CST4 measurements precludes assessment of chemotherapy-induced biomarker dynamics and their correlation with therapeutic response. Third, while the multimodal model demonstrated improved sensitivity, external validation in independent cohorts is necessary to confirm clinical applicability. Finally, the association between CST4 and PDGFRB in this study is only a correlation analysis without functional verification. In the future, animal models are needed to clarify the causal relationship and molecular mechanism between them. Future prospective multicenter studies with longitudinal sampling and standardized treatment protocols are needed to optimize CST4’s clinical utility.

## 5 Conclusion

This study establishes CST4 as a robust post-chemotherapy biomarker for CRC surveillance. Our findings demonstrate that CST4 maintains stable discriminative capacity across tumor stages and age groups, achieving superior diagnostic performance compared to conventional markers like CEA. The integration of CST4 with CEA, CA724, and CA125 into a multimodal diagnostic model significantly enhanced detection capability, overcoming limitations of single-marker approaches. Mechanistically, our *in vitro* functional experiments confirm that CST4 regulates PDGFRB expression, with CST4 knockdown leading to a significant reduction in PDGFRB levels in HCT116 cells. This validates the CST4-PDGFRB axis as a key signaling pathway involved in extracellular matrix remodeling and tumor progression, offering novel insights into CRC pathogenesis. These results position CST4 as a promising candidate for therapeutic monitoring and recurrence detection in chemotherapy-treated CRC patients, potentially addressing current gaps in post-treatment surveillance strategies.

## Data Availability

The original contributions presented in the study are included in the article/supplementary material. Further inquiries can be directed to the corresponding author.
